# Prediction of neoplastic gallbladder polyps in patients with different age level based on preoperative ultrasound: a multi-center retrospective real-world study

**DOI:** 10.1186/s12876-024-03240-9

**Published:** 2024-04-30

**Authors:** Qi Li, Minghui Dou, Hengchao Liu, Pengbo Jia, Xintuan Wang, Xilin Geng, Yu Zhang, Rui Yang, Junhui Li, Wenbin Yang, Chunhe Yao, Xiaodi Zhang, Da Lei, Chenglin Yang, Qiwei Hao, Yimin Liu, Zhihua Guo, Zhimin Geng, Dong Zhang

**Affiliations:** 1 Department of Hepatobiliary Surgery, The First Affiliated Hospital of Xi’an Jiaotong University, Xi’an, Shaanxi 710061 China; 2 Department of Hepatobiliary Surgery, The First People’s Hospital of Xianyang City, Xianyang, Shaanxi 712000 China; 3 Department of Hepatobiliary Surgery, Shaanxi Provincial People’s Hospital, Xi’an, Shaanxi 710068 China; 4 Department of Hepatobiliary Surgery, Central Hospital of Hanzhong City, Hanzhong, Shaanxi 723000 China; 5 Department of General Surgery, The Second Affiliated Hospital of Xi’an Jiaotong University, Xi’an, Shaanxi 710004 China; 6 Department of General Surgery, Xianyang Hospital of Yan’an University, Xianyang, Shaanxi 712000 China; 7 Department of General Surgery, 215 Hospital of Shaanxi Nuclear Industry, Xianyang, Shaanxi 712000 China; 8 Department of Hepatobiliary Surgery, Central Hospital of Baoji City, Baoji, Shaanxi 721000 China; 9 Department of General Surgery, Central Hospital of Ankang City, Ankang, Shaanxi 725000 China; 10 Department of Hepatobiliary Surgery, The Second Hospital of Yulin City, Yulin, Shaanxi 719000 China; 11 Department of Hepatobiliary Surgery, People’s Hospital of Baoji City, Baoji, Shaanxi 721000 China

**Keywords:** Gallbladder polyps, Neoplastic polyps, Gallbladder carcinoma, Linear scoring model

## Abstract

**Background:**

The prevalence of neoplastic polyps in gallbladder polyps (GPs) increases sharply with age and is associated with gallbladder carcinoma (GBC). This study aims to predict neoplastic polyps and provide appropriate treatment strategies based on preoperative ultrasound features in patients with different age level.

**Methods:**

According to the age classification of WHO, 1523 patients with GPs who underwent cholecystectomy from January 2015 to December 2019 at 11 tertiary hospitals in China were divided into young adults group (*n*=622), middle-aged group (*n*=665) and elderly group (*n*=236). Linear scoring models were established based on independent risk variables screened by the Logistic regression model in different age groups. The area under ROC (AUC) to evaluate the predictive ability of linear scoring models, long- and short- diameter of GPs.

**Results:**

Independent risk factors for neoplastic polyps included the number of polyps, polyp size (long diameter), and fundus in the young adults and elderly groups, while the number of polyps, polyp size (long diameter), and polyp size (short diameter) in the middle-aged groups. In different age groups, the AUCs of its linear scoring model were higher than the AUCs of the long- and short- diameter of GPs for differentiating neoplastic and non-neoplastic polyps (all *P*<0.05), and Hosmer-Lemeshow goodness of fit test showed that the prediction accuracy of the linear scoring models was higher than the long- and short- diameter of GPs (all *P*>0.05).

**Conclusion:**

The linear scoring models of the young adults, middle-aged and elderly groups can effectively distinguish neoplastic polyps from non-neoplastic polyps based on preoperative ultrasound features.

## Background

Gallbladder polyps (GPs) are common benign diseases and incidental findings from transabdominal ultrasound, which are becoming increasingly prevalent as medical imaging used widely [1, 2]. It is estimated that 0.3-9.5% of the population suffers from GPs due to differences in race, region, and so on [3, 4]. According to their pathological type, GPs are divided into non-neoplastic polyps and neoplastic polyps. Non-neoplastic polyps mainly include cholesterol polyps, inflammatory polyps, gallbladder adenomyomatosis, xanthogranuloma, adenomatous hyperplasia and so on. Neoplastic polyps can be divided into benign polyps (gallbladder adenomas and other rare benign mesenchymal tumors) and malignant polyps that mainly include gallbladder carcinoma (GBC) [5].

Currently, it has been demonstrated that the size of GPs is associated with risk, and the reported incidence of GPs transformed to GBC is 8~10% of GPs ≥10 mm, 1~3% of GPs 6 to 9 mm, and 0~0.5% of GPs <5 mm [6]. Rafaelsen et al [7] found that GPs < 6 mm had a low probability of increasing in size and none of the patients with small polyps developed GBC. Many studies have reported that GPs with a long diameter of 20 mm or larger are highly likely to be cancerous [8, 9]. According to the updated European guidelines [10, 11], cholecystectomy is recommended for GPs ≥10 mm, and GPs ≥6 mm require follow-up and cholecystectomy is recommended when combined with high risk factors of GBC (age >60 years, history of primary sclerosing cholangitis, Asian ethnicity, or sessile polypoid lesion/wall thickening >4 mm).

In addition, the prevalence of neoplastic polyps increases sharply with age and is associated with GBC [5, 12, 13]. An age criterion is also commonly conducted to guide management [10], unfortunately, the guidelines do not recommend follow-up strategies for different age groups. At present, an appropriate age threshold for recommendation remains unclear, scholars have proposed to use the age of 50-, 60- and 65- years as the thresholds for predicting malignant polyps [5]. According to the ages classification of WHO, we classified patients aged 18-44 years in the young adults group, patients aged 45-59 years in the middle-aged group, and patients aged ≥60 years in the elderly group. To identify risk factors for neoplastic polyps in patients with different age level, we analyzed the differences in clinical and ultrasound features in GPs with a long diameter of 6-20 mm. In different age groups, the study aims to predict neoplastic polyps based on preoperative ultrasound features and provide appropriate follow-up and treatment strategies.

## Methods

### Patient population

The study, involving patients with GPs undergoing cholecystectomy at 11 tertiary hospitals in China was conducted between January 2015 and December 2019. The inclusion criteria were as follows: (1) patients must be over 18 years old; (2) GPs were identified through preoperative ultrasound; (3) preoperative ultrasound indicated that the long diameter of GPs was 6-20 mm; (4) postoperative pathological examination confirmed non-neoplastic polyps or neoplastic polyps. The exclusion criteria were as follows: (1) patients with GPs combined with gallstones before cholecystectomy; (2) the long diameter of GPs was <6 mm or >20 mm; (3) preoperative diagnosis of GBC, while postoperative pathology confirmed as non-neoplastic polyps or neoplastic polyps.

### Study variables

The general indicators assessed in the study included sex, age, polyp-detection time, and digestive-system symptoms (including abdominal pain, bloating and (or) diarrhea, dyspepsia). Serological biomarkers included AST, ALT, GGT, TB, CEA, CA19-9, and CA-125. Preoperative ultrasound examination included the number of polyps, polyp size (including the long- and short- diameter, and the definitions of the long- and short- diameter of the polyps according to its size by the radiologists from each high-volume medical center), polyp site, fundus, thickness of gallbladder-wall, polyp shape, and echogenicity.

### Statistical analysis

Based on the WHO age classification, all patients from 11 tertiary hospitals were divided into young adults (*n*=622), middle-aged (*n*=665), and elderly (*n*=236) groups. Data with skewed distributions were expressed as medians (ranges), and Mann-Whitney tests were conducted to determine whether non-neoplastic polyps were different from neoplastic polyps, and the Kruskal-Wallis H test was used to analyze differences among the three age groups. The best cut-off values were determined at the maximum value of Youden index in receiver operating characteristic curves (ROC), and the areas under ROC curves (AUC) were conducted to evaluate the predictive abilities for the long- and short- diameter of GPS, respectively. The independent risk factors of neoplastic polyps were screened by χ^2^ test and the Logistic regression model for different age groups by SPSS version 25 (IBM Corp., Armonk, NY, United States). *P* < 0.05 was considered to be statistically significant.

### Establishment of linear scoring models and comparison of predictive ability

Linear scoring models were established based on independent risk variables in the young adults, middle-aged, and elderly groups. Each variable was assigned a score based on its odds ratio (OR) in the logistic regression analysis, and the total score was calculated as the sum of the subscores for different age groups [14]. For differentiating neoplastic and non-neoplastic polyps, the ROC was used to determine the best cut-off values and evaluate the predictive abilities of the linear scoring models. Based on the cut-off values, the patients were divided into low-risk and high-risk groups.

The Hosmer-Lemeshow goodness of fit was used to assess the predictive probabilities and actual probabilities for linear scoring models, long- and short- diameter of GPs in predicting neoplastic polyps, and the prediction models work well when the predictive probabilities of the prediction models match the actual probabilities when *P*>0.05; Conversely, *P*<0.05 indicates that the prediction models work poorly. The Delong test was used to compare the AUCs of the linear scoring models with the long- and short- diameter of GPs for the predictive ability of neoplastic polyps by Medcalc software (version 20.113).

## Results

### Comparison of GPs patients in the young, middle-aged and elderly groups

**Fig 1:**
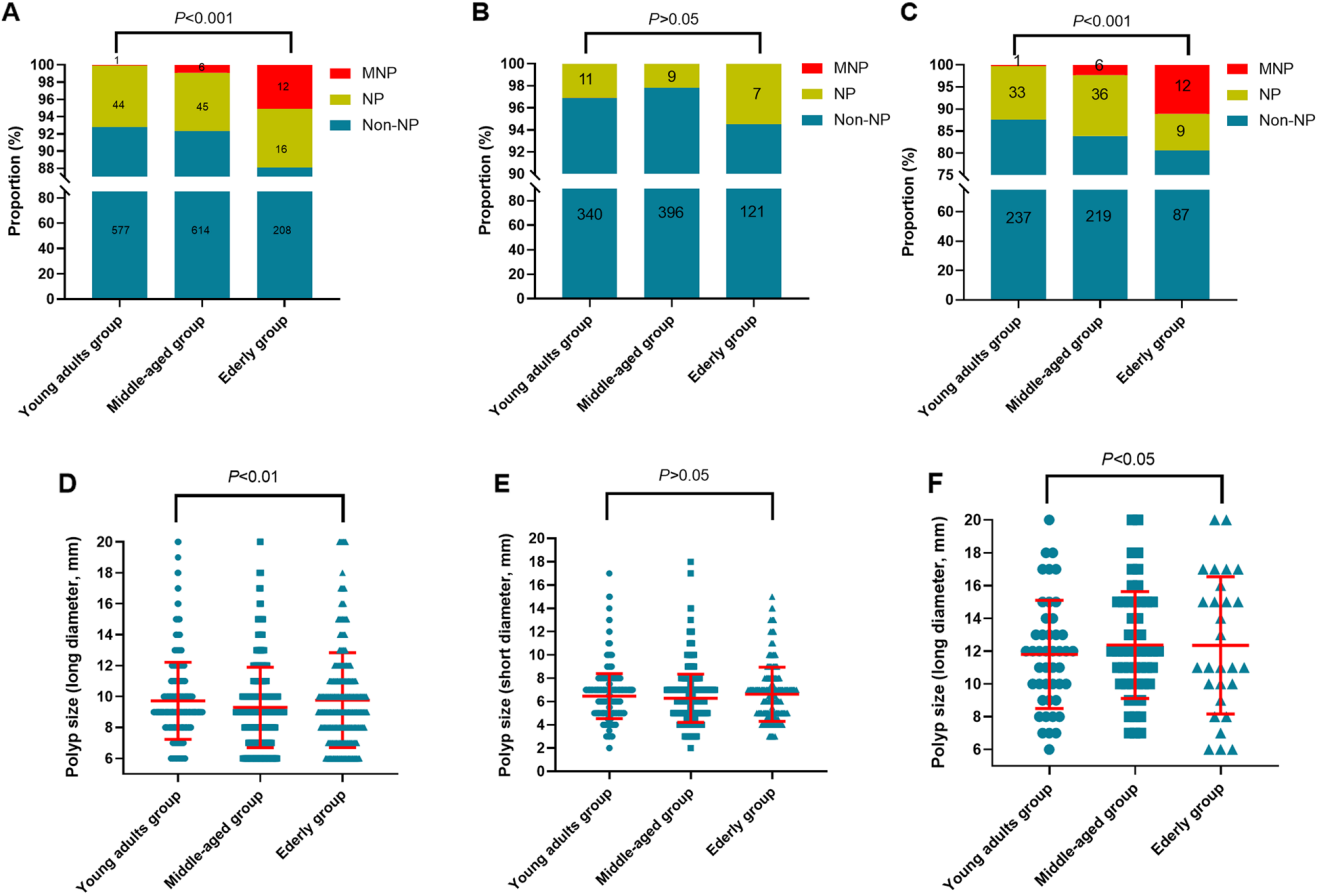
Comparison of polyp characteristics in different age groups. **A**-**C** The distribution of non-neoplastic polyps and neoplastic polyps in different age groups with the long diameter of 6-20mm, 6-9mm, and 10-20mm, respectively. **D**-**E** The distribution of the long- and short diameter of GPs in different age groups. **F** The distribution of the long diameter in neoplastic polyps in different age groups

As age increased, the proportion of neoplastic polyps increased, reaching 7.3% in young adults, 7.7% in middle-aged and 11.9% in elderly groups, of which the proportion of malignant polyps was 0.2%, 0.9% and 5.1%, respectively (Fig. 1-A, *P*<0.05). Interestingly, there were no malignant polyps in GPs patients with long diameter of 6-9 mm, and all the malignant polyps were in a long diameter of 10-20 mm. Likewise, the proportion of neoplastic polyps was significantly higher in the elderly group than in the young adults and middle-aged groups (Fig. 1-B, *P*>0.05 and Fig. 1-C, *P*<0.05). Besides, the mean values of long diameter (Fig. 1-D, *P*<0.05) and short diameter (Fig. 1-E, *P*>0.05) in the elderly group were higher than those in the young adults and middle-aged groups. The mean long diameter of neoplastic polyps in the elderly group was also higher than that in the young adults and middle-aged groups (Fig. 1-F, *P*<0.05).

By comparing the characteristics of GPs in different age groups, Sex, CEA, CA-125, number of polyps, polyp size (long diameter), polyp size (short diameter), fundus, thickness of gallbladder wall, and echogenicity were statistically different (*P<*0.05). Therefore, there were differences among different age groups in clinical and preoperative ultrasound features of GPs. Details are shown in Table 1.

**Table 1:** Comparison of preoperative clinical data of gallbladder polyps in different age groups

	Different groups (*n*=1523)			***Z/χ***^***2***^	*P*
	Young adults group (*n*=622)	Middle-aged group (*n*=665)	Elderly group (*n*=236)		
Sex					
Male	269 (43.2)	219 (32.9)	94 (39.8)	14.793	0.001
Female	353 (56.8)	446 (67.1)	142 (60.2)		
Polyp detection time					
y	244 (39.2)	307 (46.2)	101 (42.8)	9.656	0.140
1-3y	204 (32.8)	178 (26.8)	72 (30.5)		
3-5y	75 (12.1)	86 (12.9)	24 (10.2)		
y	99 (15.9)	94 (14.1)	39 (16.5)		
Combined with digestive system symptoms					
No	411 (66.1)	424 (63.8)	147 (62.3)	2.295	0.891
abdominal pain	121 (19.5)	145 (21.8)	55 (23.3)		
bloating and(or) diarrhea	67 (10.8)	68 (10.2)	25 (10.6)		
dyspepsia	23 (3.7)	29 (4.2)	9 (3.8)		
AST (U/L)	25.0 (10.0~60.0)	22.0 (10.0~60.0)	24.5 (10.0~247.0)	4.313	0.016
ALT (U/L)	31.0 (8.0~89.0)	27.0 (7.0~83.0)	30.0 (8.0~219.0)	2.513	0.285
GGT (U/L)	22.0 (1.0~177.0)	21.0 (0.0~174.0)	18.5 (0.0~245.0)	0.856	0.652
TB (μmmol/L)	10.8 (2.5~37.0)	12.3 (4.2~43.0)	11.4 (4.8~44.6)	2.091	0.351
CEA (ng/mL)	1.9 (0.0~18.6)	2.2 (0.0~9.3)	2.7 (0.3~8.6)	15.862	<0.001
CA19-9 (U/mL)	19.0 (0.6~52.6)	20.0 (0.6~125.6)	22.0 (0.1~354.0)	2.129	0.345
CA-125 (U/mL)	20.0 (1.18~63.30)	16.0 (2.5~44.0)	14.6 (1.0~145.0)	15.181	0.001
**Preoperative ultrasound feature**					
Number of polyps					
Multiple	402 (64.6)	421 (63.3)	122 (51.7)	12.952	0.002
Single	220 (35.4)	244 (36.7)	444 (48.3)		
Polyp size (long diameter)	9 (6.0~20.0)	9 (6.0~20.0)	9 (6.0~20.0)	12.181	0.002
Polyp size (short diameter)	7 (2.0~17.0)	6.0 (2.0~18.0)	6.8 (3.0~15.0)	6.480	0.039
Polyp site					
Neck	34 (5.5)	47 (7.1)	15 (6.4)	7.403	0.116
Body	442 (71.1)	470 (70.7)	150 (63.6)		
Bottom	146 (23.5)	148 (22.3)	71 (30.1)		
Fundus					
Pedicle	517 (83.1)	531 (79.8)	177 (75.0)	7.421	0.024
Broad base	105 (16.9)	134 (20.2)	59 (25.0)		
Thickness of gallbladder wall					
<4 mm	468 (75.2)	445 (66.9)	164 (69.5)	10.955	0.004
≥4 mm	154 (24.8)	220 (33.1)	72 (30.5)		
Polyp shape					
Papillary	361 (58.0)	374 (56.2)	147 (62.3)	2.737	0.603
Nodular	151 (24.30)	165 (24.8)	52 (22.0)		
Spherical and mulberry	110 (17.7)	126 (18.9)	37 (15.7)		
Echogenicity					
Low	35 (5.6)	28 (4.2)	15 (6.4)	14.394	0.006
Medium	305 (49.0)	388 (58.3)	137 (58.1)		
Strong	282 (45.3)	249 (37.4)	84 (35.6)		

### Univariate and multivariate analysis of neoplastic polyps

**Fig 2:**
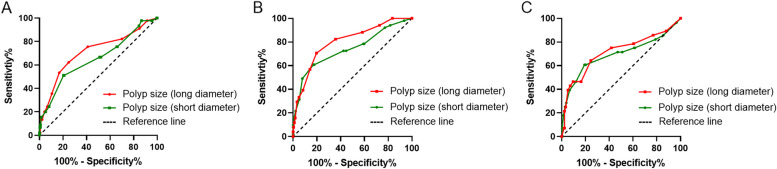
Receiver operating characteristic (ROC) curves of long- and short diameters of GPs for differentiating neoplastic and non-neoplastic polyps. **A-C** ROC curves for young adults middle-aged and elderly groups

According to the ROC curve analysis (Fig. 2 A-C), we determined the best cut-off values of long- and short- diameter of GPs were 10.5 mm and 8.0 mm in different age groups, respectively. Comparison of non-neoplastic polyps and neoplastic polyps in different age groups is shown in Table 2. Univariate analysis revealed that the number of polyps, polyp size (long diameter), polyp size (short diameter) and funds were associated with neoplastic polyps in different age groups (*P<*0.05). Multivariate analysis demonstrated that the number of polyps (single), polyp size (long diameter≥10.5 mm), and fundus (broad base) were the independent risk factors of neoplastic polyps in the young adults and elderly groups, the number of polyps (single), polyp size (long diameter≥10.5 mm), and polyp size (short diameter≥8 mm) were the independent risk factors of neoplastic polyps in the middle-aged groups.

**Table 2:**
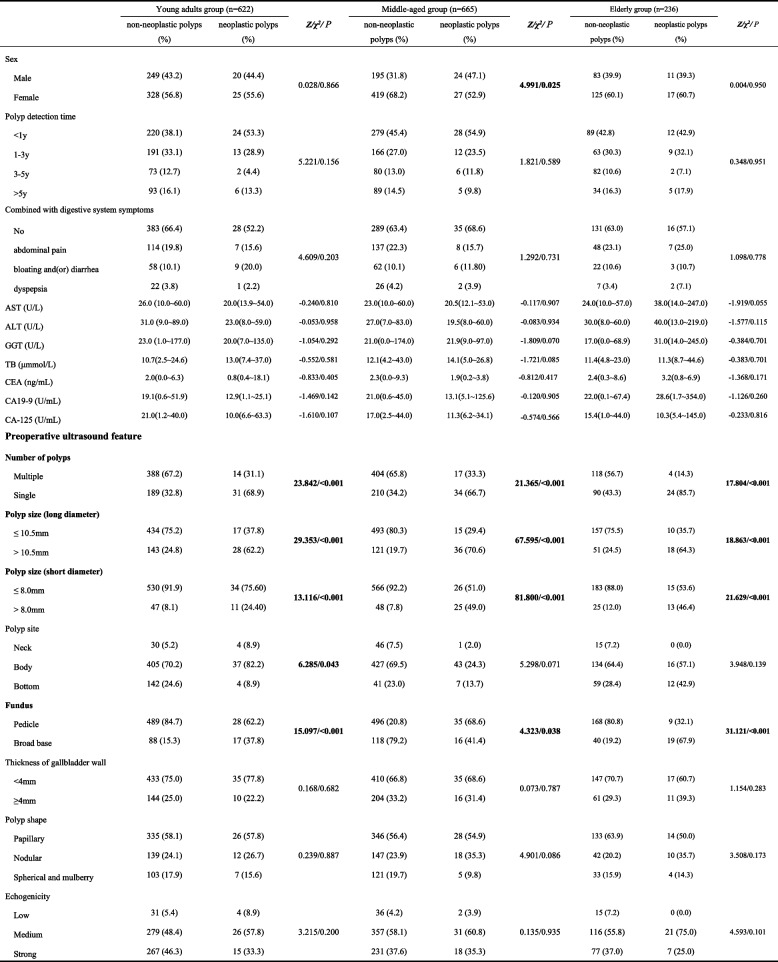
Univariate analysis for preoperative ultrasound features of 1523 cases with gallbladder polyps in different age groups

### Linear scoring models development for different age groups

Based on the independent risk factors for neoplastic polyps identified by the logistic regression model for different age groups, the linear scoring models were developed (Table 3). For the young adults group, the total score of its linear model = number of polyps (single assigned 4 points and multiple assigned 0 point) + polyp size [(long diameter), >10.5 mm assigned 4 points and ≤10.5 mm assigned 0 point] + fundus (broad base assigned 3 points and pedicle assigned 0 point). Similarly, for the middle-aged group, the total score of its linear model = number of polyps (single assigned 4 points and multiple assigned 0 point) + polyp size [(long diameter), >10.5 mm assigned 6 points and ≤10.5 mm assigned 0 point] + polyp size [(short diameter), >8 mm assigned 4 points and ≤8 mm assigned 0 point]; for the elderly group, the total score of its linear model = number of polyps (single assigned 8 points and multiple assigned 0 point) + polyp size [(long diameter), >10.5 mm assigned 6 points and ≤10.5 mm assigned 0 point] + fundus (broad base assigned 9 points and pedicle assigned 0 point).

**Table 3:** Multivariate analysis for preoperative ultrasound features of 1523 cases with gallbladder polyps in different age groups

	Young adults group (*n*=622)		Middle-aged group (*n*=665)		Elderly group (*n*=236)	
	OR (95%*CI*)	*P*	OR (95%*CI*)	*P*	OR (95%*CI*)	*P*
Number of polyps						
Single *vs.* Multiple	3.512 (1.785~6.907)	<0.001	3.576 (1.849~6.913)	<0.001	7.291 (2.343~22.055)	0.001
Polyp size (long diameter)						
*vs. *≤10.5mm	3.566 (1.843~6.901)	<0.001	5.233 (2.406~11.380)	<0.001	5.541 (2.404~12.772)	<0.001
Polyp size (short diameter)						
*vs. *≤8mm			3.641 (1.683~7.878)	0.001		
Fundus						
Broad base *vs.* Pedicle	2.404 (1.209~4.777)	0.012			8.302 (3.364~20.489)	<0.001

**Fig 3:**
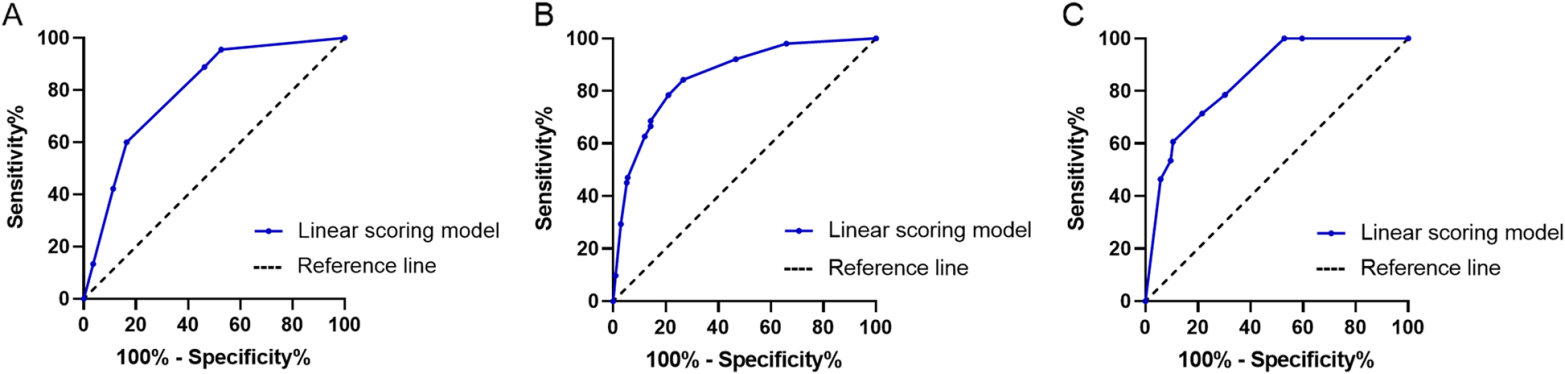
Receiver operating characteristic (ROC) curves of linear scoring models for differentiating neoplastic and non-neoplastic polyps. **A**-**C** ROC curves for young adults, middle-aged and elderly groups

According to the ROC curves analysis (Fig. 3 A-C), we determined a total score of 5.5, 5.0, and 14.5 points as the best cut-off values for differentiating neoplastic and non-neoplastic polyps, and the total score ≤ 5.5 was defined as low-risk group and >5.5 as high risk group for neoplastic polyps in the young adults group; the total score ≤5 was defined as low-risk group and >5 as high risk group for neoplastic polyps in the middle-aged group; the total score ≤ 14.5 was defined as low-risk group and >14.5 as high risk group for neoplastic polyps in the elderly group.

**Fig 4:**
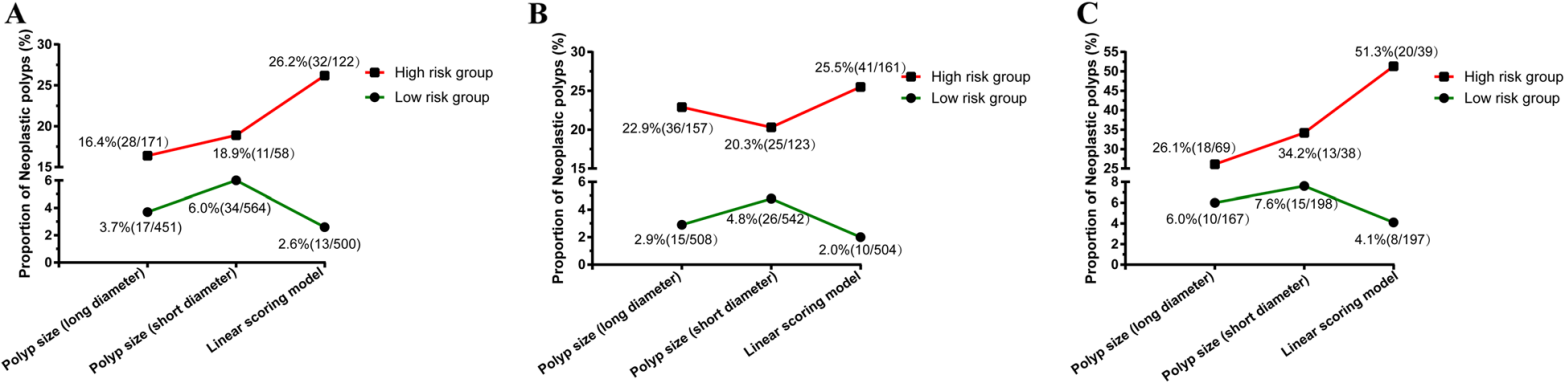
Line charts of the proportion of neoplastic polyps in low- and high-risk groups based on polyp size (long diameter), polyp size (short diameter), and linear scoring model optimal cut-off values. **A**-**C** Line charts for young adults, middle-aged and elderly groups

Compared with long diameter ≤10.5 mm and short diameter ≤8.0 mm, the proportion of neoplastic polyps in the low-risk group of the linear models decreased from 3.7% (17/451), 6.0% (34/564) to 2.6% (13/500) in the young adults group; 2.9% (15/508), 4.8% (26/542), to 2.0% (10/504) in the middle-aged group; 6.0% (10/167); 7.6% (15/198) to 4.1% (8/197) in the elderly group. Conversely, compared with long diameter > 10.5 mm and short diameter >8.0 mm and the proportion of neoplastic polyps in the high-risk group of the linear models increased from 16.4% (28/171), 18.9% (11/58) to 26.2% (32/122) in the young adults group; 22.9% (36/157), 20.3% (25/123) to 25.5% (41/161) in the middle-aged group; 26.1% (18/69), 34.2% (13/38) to 51.3% (20/39) in the elderly group (Fig. 4 A-C). Thus, cholecystectomy should be recommended for GP patients at high risk of neoplastic polyps.

### Analysis of predictive ability of linear scoring models and long- and short diameter GPs

In the young adults group, the AUC of its linear scoring model was 0.794 (95%*CI*: 0.734~0.853), which was higher than the AUCs of the long diameter (AUC: 0.712, 95%*CI*: 0.624~0.800) and short diameter (AUC: 0.654, 95%*CI*: 0.562~0.746) of GPs for differentiating neoplastic and non-neoplastic polyps (all *P*<0.05). Similarly, in the middle-aged group, the AUC of its linear scoring model was 0.857 (95%*CI*: 0.805~0.908), which was higher than the AUCs of the long diameter (AUC: 0.802, 95%*CI*: 0.738~0.867) and short diameter (AUC: 0.740, 95%*CI*: 0.657~0.823) of GPs (all *P*<0.05); in the elderly group, the AUC of its linear scoring model was 0.849 (95%*CI*: 0.784~0.914), which was higher than the AUCs of the long diameter (AUC: 0.714, 95%*CI*: 0.593~0.835) and short diameter (AUC: 0.693, 95%*CI*: 0.564~0.822) of GPs (all *P*<0.05).

Hosmer-Lemeshow goodness of fit test indicated that it was more acceptable for the predictive probabilities of the linear scoring models than the long- and short diameter of GPs to fit the actual probabilities (all* P*>0.05), which showed that the prediction accuracy of the linear scoring models in different age groups was higher than the long- and short diameter of GPs for predicting the neoplastic polyps. Details are shown in Table 4.

**Table 4:** Comparison of the predictive ability of different models to predict neoplastic polyps

Groups	Different prediction models	ROC				Delong test		Hosmer-Lemeshow goodness of fit	
		AUC (95%CI)	Sensitivity (%)	Specificity (%)	*P*	Z value	*P*	***χ***^***2***^	*P*
Young adults group (*n*=622)	Polyp size (long diameter)	0.712(0.624~0.800)	62.2	75.2	< 0.001	2.062	< 0.05	9.798	0.133
	Polyp size (short diameter)	0.654 (0.562~0.746)	51.1	79.5	0.001	2.856	< 0.01	13.576	< 0.05
	Linear scoring model	**0.794 (0.734~0.853)**	**60.0**	**83.5**	**< 0.001**	**Ref**		**4.440**	**0.221**
Middle-age group (*n*=665)	Polyp size (long diameter)	0.802 (0.738~0.867)	70.6	80.3	< 0.001	2.208	< 0.05	10.311	0.067
	Polyp size (short diameter)	0.740 (0.657~0.823)	60.8	82.9	< 0.001	3.516	< 0.001	10.343	0.170
	Linear scoring model	**0.857 (0.805~0.908)**	**84.3**	**73.3**	**< 0.001**	**Ref**		**1.512**	**0.825**
Elderly group (*n*=236)	Polyp size (long diameter)	0.714 (0.593~0.835)	64.3	75.5	< 0.001	3.037	< 0.01	9.974	0.126
	Polyp size (short diameter)	0.693 (0.564~0.822)	60.7	80.8	0.001	2.869	< 0.01	9.432	0.223
	Linear scoring model	**0.849 (0.784~0.914)**	**60.7**	**89.4**	**< 0.001**	**Ref**		**5.041**	**0.411**

## Discussion

While GBC is an uncommon malignancy, its prognosis is extremely poor and varies according to the stage of the disease at the time of surgery, with a 5-year survival rate of around 10% to 50% [15]. In order to reduce the incidence of GBC, identifying gallbladder precancerous lesions, improving the early diagnosis rate, and undergoing cholecystectomy in time are of great importance. Currently, many international guidelines use a size criterion to guide patient management [10, 16, 17]. However, there is controversy regarding the threshold for GPs to recommend cholecystectomy based on the long diameter [13, 18–20]. In this study, for GPs with long diameter of 6-20mm, we determined the best cut-off values of long- and short diameter of GPs as 10.5 mm and 8.0 mm in different age groups, respectively.

By comparing the characteristics of GPs in different age groups, we found a statistically significant difference in number of polyps, polyp size (long diameter), polyp size (short diameter), fundus, thickness of gallbladder wall, and echogenicity. As a result, it is very important that GPs establish their own follow-up and treatment strategies for patients in different age level. In multivariate analysis, the number of polyps, polyp size (long diameter), polyp size (short diameter), and fundus were independent risk factors, which are consistent with the surgical indications recommended by the European guidelines [10]. Thus, establishing predictive models for neoplastic gallbladder polyps based on the independent variables is of great importance to prevent GBC and avoid unnecessary cholecystectomy.

By utilizing the Logistic regression model for different age groups, we developed linear scoring models were developed based on the independent risk factors of neoplastic polyps, which had the advantage of being easy to apply in clinical settings. In different age groups, the AUCs of its linear scoring model were higher than that of the long- and short diameter of GPs for differentiating neoplastic and non-neoplastic polyps (all *P*<0.05). Therefore, establishing predictive models based on independent risk factors can effectively improve the diagnostic ability. At present, the prediction models developed for neoplastic polyps or GPs with malignant tendency were general population models, and there are no prediction models for neoplastic polyps in the young adults patients or neoplastic polyps in elderly patients [12, 21–23]. We previously established a nomogram prediction model for long diameter10-15 mm GPs with malignant tendency, but did not provide follow-up and treatment strategies for different age groups [24]. Thus, we try to propose follow-up and treatment strategies for the young adults, middle-aged and elderly individuals.

At present, several studies have established linear scoring models for all populations to identify neoplastic polyps or polyps with malignant propensity. Ma et al [14] established a linear scoring system based on age, positive blood flow signal, and cross-sectional area of GPs for identifying true polyps, which were accurate with an AUC of 0.883. Güneş et al [25] established a linear scoring system based on age ≥50, presence of symptoms, polyp size >12.5 mm, single polyp, concomitant gallstones, and gallbladder wall thickness ≥4mm for identifying the GPs with malignant potential with an AUC of 0.958 for risk scores. In addition, Yuan et al [26] established an ultrasound radiomic model based on the spatial and morphological features extracted from ultrasound images, which effectively contributed to the preoperative diagnosis of true and pseudo GPs with an AUC of 0.898. Although the AUCs of our linear scoring models for different age groups were slightly lower than the above studies [14, 25, 26], we consider the variables included in our study to be relatively less compared to their studies.

Meanwhile, according to the ROC curve analysis, we observed that compared to the long- and short- diameter of GPs, the low-risk group of the linear scoring models of different age groups could decrease the proportion of neoplastic polyps, while the high-risk group could identify a higher proportion of neoplastic polyps. In clinical practice, cholecystectomy should be recommended for high-risk groups based on our linear scoring models in different age groups, and regular follow-up should be recommended for low-risk groups.

Several limitations are present in this study. We developed simple linear scoring models based on independent risk factors for patients with neoplastic polyps in different age groups, which may provide less predictive power than nomograms and other models established by machine learning algorithms. Additionally, the linear scoring models were established based on only three out of four variables such as the number of polyps, polyp size (long diameter), polyp size (short diameter) or funds. The characterization of neoplastic polyps is crucial to determining the precise treatment of GPs and preventing GBC in different age groups. Accordingly, here is still a need for more extensive datasets from additional medical centers for internal or external validation, including larger samples with a wider variety of clinical variables, particularly serological biomarkers and preoperative imaging features. Future developments should focus on the establishment of predictive models with higher accuracy and predictive capabilities based on various machine learning methods.

## Conclusion

To conclude, three linear scoring models showed superior predictions of neoplastic polyps compared with GPs with long- and short- diameters, which provided important reference values and guidance for GPs regarding whether to recommend cholecystectomy for patients. Therefore, linear scoring models based on preoperative ultrasound features from young adults, middle-aged, and elderly groups can be used to discriminate between neoplastic polyps and non-neoplastic polyps.

## Data Availability

The data used in this study is not a public data. The datasets used in this study are available from the corresponding authors on reasonable requests.

## Abbreviations

ALT: Alanine transaminase
AST: Aspartate transaminase
AUC: The area under ROC
CA125: Carbohydrate antigen 125
CA19-9: Carbohydrate antigen 19-9
CEA: Carcinoembryonic antigen
GBC: Gallbladder carcinoma
GGT: Gamma-glutamyl transpeptidase
GPs: Gallbladder polyps
OR: Odds ratio
TB: Total bilirubin
ROC: Receiver operating characteristic curve
